# Institute collection and analysis of Nanobodies (iCAN): a comprehensive database and analysis platform for nanobodies

**DOI:** 10.1186/s12864-017-4204-6

**Published:** 2017-10-17

**Authors:** Jing Zuo, Jian Li, Rongxin Zhang, Longsheng Xu, Hanhan Chen, Xiaohuan Jia, Zhipeng Su, Linhong Zhao, Xing Huang, Wei Xie

**Affiliations:** The Key Laboratory of Developmental Genes and Human Disease, Ministry of Education, Institute of Life Sciences, Southeast University, Nanjing, China

**Keywords:** Nanobody, Nanobody sequence analysis, Database

## Abstract

**Background:**

Nanobodies are single-domain antibodies that contain the unique structural and functional properties of naturally-occurring heavy chain in camelidae. As a novel class of antibody, they show many advantages compared with traditional antibodies such as smaller size, higher stability, improved specificity, more easily expressed in microorganisms. These unusual hallmarks make them as promising tools in basic research and clinical practice. Although thousands of nanobodies are known to be published, no single database provides searchable, unified annotation and integrative analysis tools for these various nanobodies.

**Results:**

Here, we present the database of Institute Collection and Analysis of Nanobodies (iCAN). It is built for the aim that addressing the above gap to expand and accelerate the nanobody research. iCAN, as the first database of nanobody, contains the most comprehensive information to date on nanobodies and related antigens. So far, iCAN incorporates 2391 entries which include 2131 from patents and 260 from publications and provides a simple user interface for researchers to retrieve and view the detailed information of nanobodies. In addition to the data collection, iCAN also provides online bioinformatic tools for sequence analysis and characteristic feature extraction.

**Conclusions:**

In summary, iCAN enables researchers to analyze nanobody features and explore the applications of nanobodies more efficiently. iCAN is freely available at http://ican.ils.seu.edu.cn.

**Electronic supplementary material:**

The online version of this article (10.1186/s12864-017-4204-6) contains supplementary material, which is available to authorized users.

## Background

Camelidae (*Camelus ferus*, *Camelus bactrianus*, *Camelus dromedarius*, *Vicugna vicugna*, *Vicugna pacos*, *Lama guanicoe*, *Lama glama*) and some cartilaginous fish (nurse shark, wobbegong and dogfish sharks) were discovered to produce functional antibodies which naturally lacked light chains [1, 2]. The single domain antigen-binding fragments of camelid heavy chain antibodies are referred to VHHs or nanobodies. Nanobody entity contains four framework regions (FR1–4), forming the core structure of the domain, which are alternated with three complementary determining regions (CDR1–3). Compared with traditional antibodies, nanobodies show obvious advantages: higher affinity, higher solubility, higher domain stability, smaller size (~15 kda) and recognition of hidden antigenic sites, reduced aggregation tendencies, more easily expressed in microorganisms [3–8].

In the latest years, there has been an enormous growth of interest in studying nanobody for its unique characteristic structure and being the ideal candidate for the development of sophisticated nanobiotechnologies in diverse fields. In basic research, nanobodies are developed into various research tools such as affinity purification [9], gene activation or inactivation [10], immunoprecipitation [11]. Due to the nanoscopic size and high affinity against intracellular signaling molecules, nanobodies and their derivative formats are used as nanotracer in intracellular bioimaging [12]. In addition, nanobodies are applied in disease diagnosis, for example, molecular diagnosis for breast cancer [13]. The application of nanobodies in targeting therapeutics is in an early stage but shows good prospects in the therapy of acute thrombotic thrombocytopenic purpura [14], infectious disease [15], rheumatoid arthritis [16], central nervous system disease [17], breast and ovarian cancers [18] and so on. Besides, nanobodies are used to be enzyme inhibitors to increase starch content [19] and also to do food testing in agriculture.

Currently, the application research of nanobody is still in exploration. Although thousands of nanobodies have been published, there exists no database of nanobodies integrating nanobody resources. iCAN is built as the first comprehensive nanobody database, which is presented for the aim that providing searchable, unified annotation and integrative analysis tools for both academic and industrial researchers to expand and accelerate the nanobody research. In order to accomplish this, the annotation data was collected and integrated from public sources. The first version of iCAN has 2391 entries, comprising 74 nanobodies from RCSB PDB, 156 nanobodies from EMBL and 2131 nanobodies from patents. Information related to nanobody sequence, structure, target antigens, function, taxonomy of the source organism is shown. In addition, iCAN also provides online bioinformatic tools which are designed for sequence analysis such as Blast, Clustal Omega, Motif extraction and CDR prediction tool. These tools can be utilized to fast discover characteristic feature of nanobodies against one special antigen in sequence. Altogether, iCAN will aid the scientific/industrial community in analyzing the nanobody features and exploring the applications of nanobodies.

## Construction and content

### Data collection and organization

Sequences and structural information of nanobodies were retrieved from databases of RCSB PDB (http://www.rcsb.org/pdb/home/home.do), EMBL (http://www.embl.org/), PubMed (https://www.ncbi.nlm.nih.gov/pubmed) and public patents using combination of keywords like ‘nanobody’, ‘single domain antibody’, and ‘VHH’. Information on nanobody sequence, structure, targeted antigen, source organism and publication details was manually curated. Currently, iCAN includes curated and unified annotations of 2391 unique nanobody entries. The structure information of 74 nanobodies is shown in Structure interface separately. Because there is no a nomenclature for nanobodies until now, we renamed all curated nanobodies following a format ‘CAN_serial number’ as their temporary names and unique identifiers in our database.

### Database architecture

**Fig. 1:**
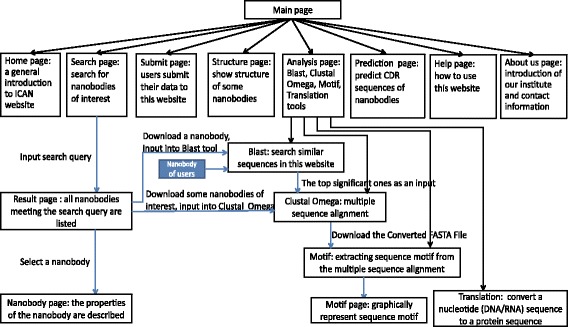
The architecture of iCAN website. iCAN website includes eight interfaces and five frequently used tools. The *blue* line represents the information flow

iCAN is a user-friendly website with concise interfaces which enable easy and fast navigation. Figure 1 illustrates the architecture of this database site. The detailed introduction to website interfaces can be viewed in the Help page of iCAN or the web manual in Additional file 1.

iCAN is architected to provide a unified resource for the scientific/industrial community allowing users to locate items in this database by any of these queries: (1) list the related information such as targeted antigen, ‘CAN_serial number’ of nanobodies, function and source organism; (2) query by raw sequence such as nanobody full length sequence or CDR1, 2, 3 domain sequence; (3) list the identifier of published origin like PDB ID, PubMed ID. Besides, users can view structure information through PDB link after screening nanobodies of interest in Structure interface (Additional file 2: Figure S1a). iCAN is also architected to aid researchers to do sequence analysis with four frequently-used tools (Blast, Clustal Omega, Translation and Motif) in Analysis interface and CDR prediction tool in Prediction interface. In order to collect as many nanobodies as possible to achieve the aim of nanobody prediction in the future, iCAN provides Submit interface (Additional file 2: Figure S1b) for researchers to upload their own nanobodies.

### Database implementation

iCAN is built on Apache HTTP Server (V2.4.43) with PHP (V5.6.24). The back-end functions using MySQL Server (V5.5.53) and the front-end works using Cascading Style Sheets (CSS), HyperText Markup Language (HTML). The required HTML pages are returned in response to user query. After the user’s Web browser sends a HTTP query to Web server, the required script including database query is executed. Then the PHP script dynamically generates results in the form of an HTML page and sends to the user’s computer (Additional file 3: Figure S2).

### Statistical results

**Fig. 2:**
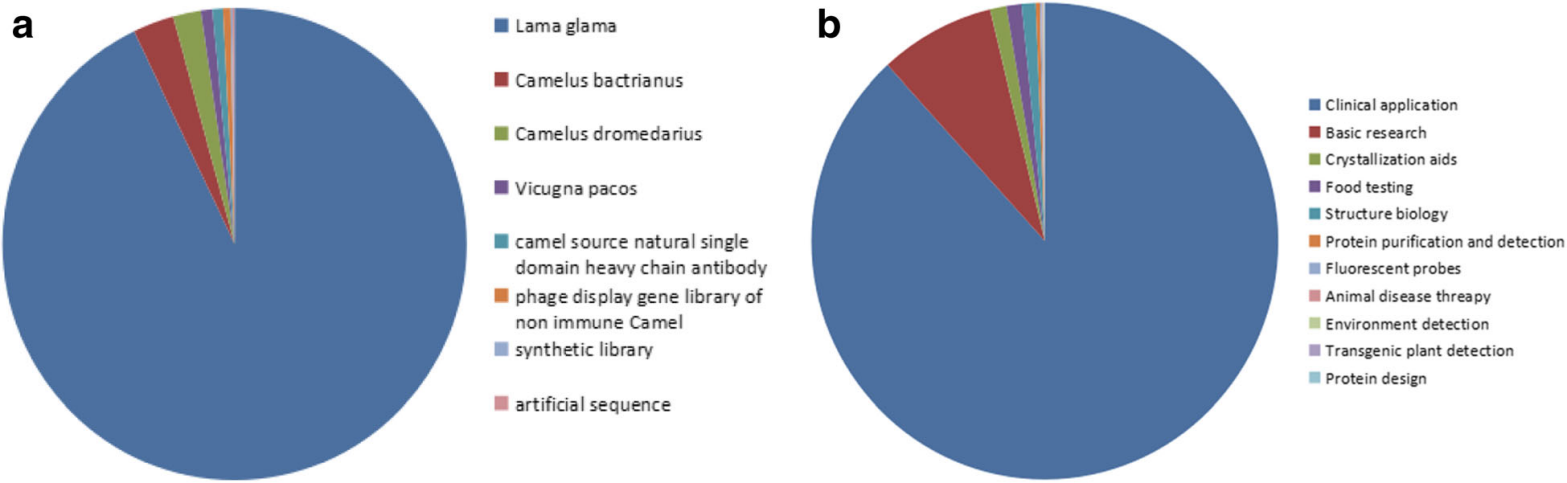
The pie chart of nanobody’s source organism and applicaiton. **a** The taxonomy of nanobody’s source organism in iCAN. The proportion of nanobodies’ source organisms is shown in different colors. The unknown are not included in this chart. **b** The applications of nanobodies in iCAN database. The related proportion of different applications is shown. The unknown are not included in this chart

The iCAN database currently contains 2391 nanobodies (Additional file 4). The source organism of nanobody is mainly *Lama glama* (1395), *Camelus bactrianus* (48) and *Camelus dromedarius* (27). Most nanobodies are obtained by immunizing animals, whereas, some nanobodies are yielded through phage screening natural antibody repertoires from non-immunized camels or from synthesized library (Fig. 2a). Recently, nanobodies are mostly applied in clinical practice (1863), basic research (130). Besides, they are also used in fields of crystallization aids, food testing and structure biology (Fig. 2b).

## Utility

Obtaining detailed information and sequence feature for nanobody of interest is necessary for nanobody research and application. iCAN provides users with such information and convenience. Here we present an example that one can get the sequence feature of nanobodies for a given antigen (Human Epidermal Growth Factor Receptor, EGFR) by using iCAN data and analytic tools. To achieve the above goal, one can follow the below pipeline: obtaining nanobody sequence by searching antigen name or by blasting a given similar nanobody sequence, followed by downloading the data, multiple sequence alignment and motif extraction.

### Obtaining nanobody information by searching

Basic search and advanced search are very useful functions, which were developed to identify nanobody based on name, antigen, PDB ID, function, source organism, etc. The detailed information about nanobodies can be viewed by clicking on search result that links to general information page. For example, we get 68 entries of the resulting nanobodies using Basic Search by typing EGFR as antigen.

### Obtaining nanobody sequence by BLAST

**Fig. 3:**
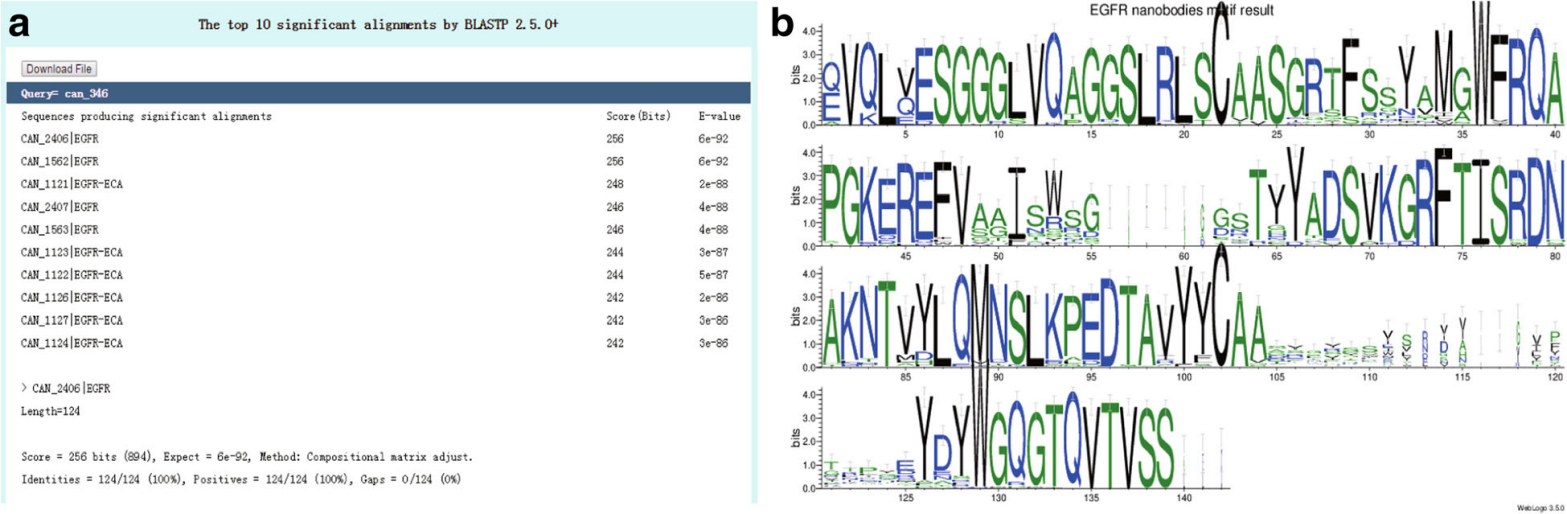
The example result of Blast and Motif tool. **a** The example result of Blast in iCAN. The top 10 significant alignments and related E-values are shown. **b** The example result of Motif tool in iCAN. One stack represents one position in the sequence. The sequence conservation at each position can be seen from the overall height of each stack, while the relative frequency of each amino acid at that position is indicated by the height of symbols within the stack

BLAST is one of the most important tools for searching nanobodies with sequence as the keyword. It can be used to search nanobody from all nanobodies or, specially, from the patented nanobodies recorded in iCAN database. In the page of Blast search result, the top 10 significant alignments are shown (Fig. 3a).

### Data download

Users can download the search results in FASTA format in a batch which will be used in multiple sequence alignments.

### Multiple sequence alignments browsing

The nanobody sequence alignments can be viewed by Clustal Omega. We input the downloaded results to Clustal Omega module and then download the converted FASTA file.

### Sequence motif extraction

The downloaded converted FASTA file from the last step is used as input data for extracting sequence motif. A graphical result of sequence motif feature from the multiple sequence alignment is shown in Fig. 3b. We can get the primary information about each position of nanobody sequence and corresponding proportion of EGFR nanobodies.

## Discussion

Besides our current work, there are still many aspects of the database that can be further improved in the future. First, a large fraction of nanobody sequences are likely not to be published for some reasons such as commercial interest, which are not included in this database. It is very necessary to expand the number of nanobodies of iCAN in the future with the help of all researchers. Second, structure of nanobody is key feature for specially recognizing and binding with antigen, thus exploration of nanobody structure is important for designing novel functional nanobody for target antigen. In the current version of the database, the structure data of many nanobodies is vacant. More structure data are required. As fast increased nanobody structure data will be uploaded to PDB database, more nanobody 3D structure data will be collected and help us get an idea about the relationship between nanobody sequence and its 3D structure.

Additionally, as nanobody has family-specific sequence composition, with fast increasing sequence data of nanobodies, there is a possibility that one can mine the sequence to discover nanobody sequence feature and try to de novo design novel nanobody sequence. We will further build-up and embed protein homology modeling tools in our database to describe nanobody structure feature. The above work will be completed with more accumulation of nanobody sequence and novel tools involvement. At that time, iCAN will provide more powerful functions for users.

## Conclusions

In this study, we present the first database of nanobody, iCAN. That is a comprehensive and free database of nanobody which not only collects the nanobody information, but also provides tools for sequence alignment, pattern feature creation and keywords-based search function. The information and tools in iCAN will enable researchers to work on nanobody more efficiently.

## Availability and requirements

The datasets generated and/or analyzed during the current study are available in the iCAN repository, http://ican.ils.seu.edu.cn

Browser requirement: we recommend the use of the IE, Chrome or Firefox web browsers for an optional experience.

Datasets in iCAN are freely available for academic users. For all other uses, please contact with us by email: ican@seu.edu.cn

## Additional files

Additional file 1: iCAN web manual.doc: the manual of iCAN website. (DOCX 91 kb)

Additional file 2: Structure and Submit pages of iCAN website. (a) Structure page of iCAN (b) Submit page of iCAN. (PDF 639 kb)

Additional file 3: Figure S1. The workflow of iCAN website. After the user’s Web browser sends a HTTP query, the required HTML pages are returned in response to user’s query. (PDF 294 kb)

Additional file 4: Figure S2. all data.xls: the source data of all curated nanobody. (XLS 2544 kb)

## Abbreviations

CDR: Complementarity determining region
CSS: Cascading style sheets
EGFR: Human epidermal growth factor receptor
FR: Framework region
HTML: Hyper text markup language
iCAN: Institute collection and analysis of nanobodies
