# Supplementary material for: Institute collection and analysis of Nanobodies (iCAN): a comprehensive database and analysis platform for nanobodies
Source: BMC Genomics. 2017 Oct 17;18:797. doi: 10.1186/s12864-017-4204-6 (PMC5646159; doi:10.1186/s12864-017-4204-6)
Supplement: Supplementary file 1 — iCAN web manual.doc: the manual of iCAN website. (DOCX 91 kb) [file 12864_2017_4204_MOESM1_ESM.docx]

**iCAN: Institute Collection and Analysis of Nanobodies**

**User’s manual**

**Introduction**

Nanobodies are single-domain antibodies derived from the variable regions of camelidae atypical immunoglobulins (Igs). They are highly valued as high-affinity reagents for research, diagnostics and therapeutics owing to their high specificity, small size (~15 kda) and straightforward bacterial expression. Nanobodies are now being studied for use in various disease areas, including oncology, infectious, inflammatory, and neurodegenerative diseases. It is generally recognized that nanobodies have extensive application prospects in diagnosis and therapy in the future.

iCAN has been created with an objective to prospect the academic research and clinical application of nanobodies. To our knowledge, it is the first comprehensive database of nanobody. This manually curated database currently holds 2391 nanobody sequences including 74 nanobodies from RCSB PDB, 156 nanobodies from EMBL, and 2131 patented nanobodies from patents. Information related to nanobody DNA sequence, protein sequence, structure, target antigens, function, taxonomy of source organism are provided. Frequently used tools such as CDR prediction tool, Blast and Clustal Omega are included here. The website also provides functions of sequence upload and analysis. The database will be updated monthly with additional nanobodies.

**Search**

We classified search into basic search and advanced search in iCAN. Basic search allows users to search based on keywords like nanobody name, antigen, PDB ID, function, PubMed ID, source organism, etc. Advanced search allows users to restrict the search to a combination of varied feature description. The detailed information about nanobodies can be viewed by clicking on search result that links to general information page. Related Links to Pubmed and patent website is provided for every entry. Meanwhile, the CDR domains are highlighted in the sequence.

Both searches are case insensitive. We have added an example in searching section. A complete list of the field descriptors and their description is given below:

| DESCRIPTORS | DESCRIPTION |
| --- | --- |
| Antigen | The name of an antigen, e.g. GFP |
| PDB ID | PDB entry name, e.g. 2X1O |
| Name | The name of nanobodies in iCAN, e.g. CAN_002 |
| PubMed ID | PubMed entry number, e.g. 23911607 |
| Function | The function of nanobodies, e.g. Food testing |
| Source organism | The animal source of the nanobodies, e.g. Lama glama |
| Bacteria family | The bacteria family for expression of nanobodies, such as E.coli TG1 |

**Analysis**

The analysis interface provides four frequently-used tools for sequence analysis.

**BLAST**

BLAST, namely Basic Local Alignment Search Tool, is a sequence comparative tool, which is used to find local similar regions between sequences. Users can use it to compare protein or nucleotide sequences to chosen sequence databases and obtain the statistical results of matches that can help users judge the confidence of the alignment. This search tool allows scientists to infer the function of a sequence referring to similar sequences. It also can be used to infer evolutionary relationships between sequences and help identify family members.

BLAST in iCAN allows users to choose databases of interest such as the entire database and the only patented nanobody database.

***How to use this tool?***

Step-1 Enter Query Sequence

Users should enter query sequence in FASTA format directly into the input data box.

Step-2 Set parameters

Default parameter choices are set for the intended uses of the tools. Users can adjust them according to their need.

E-value

The Expect value (E) is a parameter that describes the number of hits one can "expect" to see by chance when searching a database of a particular size. The lower the E-value, or the closer it is to zero, the more "significant" the match is. When the Expect value is increased from the default value of 10, a larger list with more low-scoring hits can be reported.

*Default value is: 10*

Matrix

The "substitution matrix" is a key element in evaluating the quality of a pairwise sequence alignment, which assigns a score for aligning any possible pair of residues. Users can select the scoring matrix according to the feature of sequences and their need.

*Default value is: BLOSUM62*

Databases

Users can choose the comparative databases of interest.

*Default value is: ALL*

Alignment View

Choose the alignment view, pairwise or multiple.

*Default value is: pairwise*

Step-3 Submission

**References**

- Gibrat JF, Madej T, Bryant SH. Surprising similarities in structure comparison. Curr Opin Struct Biol. 1996 Jun; 6(3): 377-85.

*FASTA format*

FASTA format for sequences begins with a single-line description, followed by lines of sequence data. The description line is demarked from the sequence data by a greater-than ('>') symbol in the first column.

For example:

>ENA|AJ238057|AJ238057.1 Lama glama partial mRNA for immunoglobulin heavy chain variable region (IGHV gene), clone WH25

CTGCAGGAGTCAGGGGGAGGCTTGGTGCAGCCTGGGGGGTCTCTGAAACTCTCCTGTGCG

**Clustal Omega**
Clustal Omega is a tool for multiple sequence alignment. It is the latest addition to the Clustal family. It can align hundreds of thousands of sequences quickly and deliver accurate alignments because of the new HMM alignment engine. Users can paste their sequences in the FASTA format. After alignment, users can see evolutionary relationships via viewing Cladograms or Phylograms which are beneficial for discovering and designing novel nanobody sequence.

***How to use this tool?***

Step 1 - Sequence

The first step is to set the tool input. Users can input sequences directly or upload sequence files.

Sequence Input Window

Users can directly enter three or more sequences to be aligned into the input box. Sequences should be in FASTA format. A return should be added to the end of the sequence to help certain applications understand the input. Note that Word processor files or data from Word processor may lead to unpredictable results as hidden/control characters may be present in the files. There is currently a limit of 2000 sequences.

Step 2 - Set Your Parameters

Default parameter choices are set for the intended uses of the tools, and can be adjusted by the tool user.

Output Alignment Format

Format for generated multiple sequence alignment.

| **Option** | **Description** | **Abbreviation** |
| --- | --- | --- |
| CLUSTAL | Clustal alignment format without base/residue numbering | clu |
| FASTA | FASTA sequence format | fa |
| MSF | Multiple Sequence File (MSF) alignment format | msf |
| PHYLIP | PHYLIP interleaved alignment format | phy |
| SELEX | SELEX alignment format | selex |
| STOCKHOLM | STOCKHOLM alignment format | st |
| VIENNA | VIENNA alignment format | vie |

*Default value is: FASTA[fa]*

Dealign Input Sequences

Remove any existing alignment (gaps) from input sequences.

| **Option** | **Description** | **Abbreviation** |
| --- | --- | --- |
| no |  | false |
| yes |  | true |

*Default value is: no [false]*

Number of Combined Iterations

Number of (combined guide-tree/HMM) iterations.

*Default value is: default (0)*

Max Guide Tree Iterations

Having set the number of combined iterations, this parameter can be changed to limit the number of guide tree iterations within the combined iterations.

*Default value is: default*

Max HMM Iterations

Having set the number of combined iterations, this parameter can be changed to limit the number of HMM iterations within the combined iterations.

*Default value is: default*

Step 3 – Submission

**References**

- Sievers F., Wilm A., Dineen D., Gibson T.J., Karplus K., Li W., Lopez R., McWilliam H., Remmert M., Söding J., Thompson J.D., Higgins D.G. (2011) Fast, scalable generation of high-quality protein multiple sequence alignments using Clustal Omega. Mol Syst Biol. 2011 Oct 11;7:539. doi: 10.1038/msb.2011.75.
- Goujon M., McWilliam H., Li W., Valentin F., Squizzato S., Paern J., Lopez R. (2010) A new bioinformatics analysis tools framework at EMBL-EBI. Nucleic Acids Res. 2010 Jul;38(Web Server issue):W695-9. doi: 10.1093/nar/gkq313. Epub 2010 May 3.
- McWilliam H., Li W., Uludag M., Squizzato S., Park Y.M., Buso N., Cowley A.P., Lopez R.(2013) Analysis Tool Web Services from the EMBL-EBI. Nucleic Acids Res. 2013 Jul;41(Web Server issue):W597-600. doi: 10.1093/nar/gkt376. Epub 2013 May 13. [[](#_ENREF_1)1] [(Trong et al., 201](#_ENREF_1)3) [[](#_ENREF_1)1]

**Motif**

“Motif” is a short conserved region in a protein sequence. This tool graphically represents amino acids or nucleic acids multiple sequence alignment. Each chart consists of stacks of symbols, of which one stack represents one position in the sequence. The sequence conservation at each position can be seen from the overall height of each stack, while the relative frequency of each amino or nucleic acid at that position is indicated by the height of symbols within the stack. The width of the stack is proportional to the fraction of valid symbols in that position. (Positions with many gaps have thin stacks). The stacks display the colors that are chosen according to the chemical species they represent. The default colors for nucleotides are G, orange; T and U, red; C, blue; and A, green. Amino acids have colors according to their chemical properties, that is to say, polar amino acids (G, S, T, Y, C, Q, N) show as green, basic (K, R, H) blue, acidic (D, E) red, and hydrophobic (A,V, L, I, P, W, F, M) amino acids as black.

The Motif tool can be used to discover sequence feature for a given group of nanobodies of interest, by which users can find functional domains and design novel nanobody sequence.

**References**

- Timothy L. Bailey and Charles Elkan, "Fitting a mixture model by expectation maximization to discover motifs in biopolymers", Proceedings of the Second International Conference on Intelligent Systems for Molecular Biology, pp. 28-36, AAAI Press, Menlo Park, California, 1994.
- [Crooks GE](http://threeplusone.com/), [Hon G](http://compbio.berkeley.edu/), [Chandonia JM](http://compbio.berkeley.edu/), [Brenner SE](http://compbio.berkeley.edu/people/brenner/) WebLogo: A sequence logo generator, Genome Research, 14:1188-1190, (2004)
- Schneider TD, Stephens RM. 1990.[Sequence Logos: A New Way to Display Consensus Sequences.](http://www.lecb.ncifcrf.gov/~toms/paper/logopaper/)Nucleic Acids Res.18:6097-6100

**A pipeline of tandem utilization of Clustal Omega and Motif tool modules:**

**
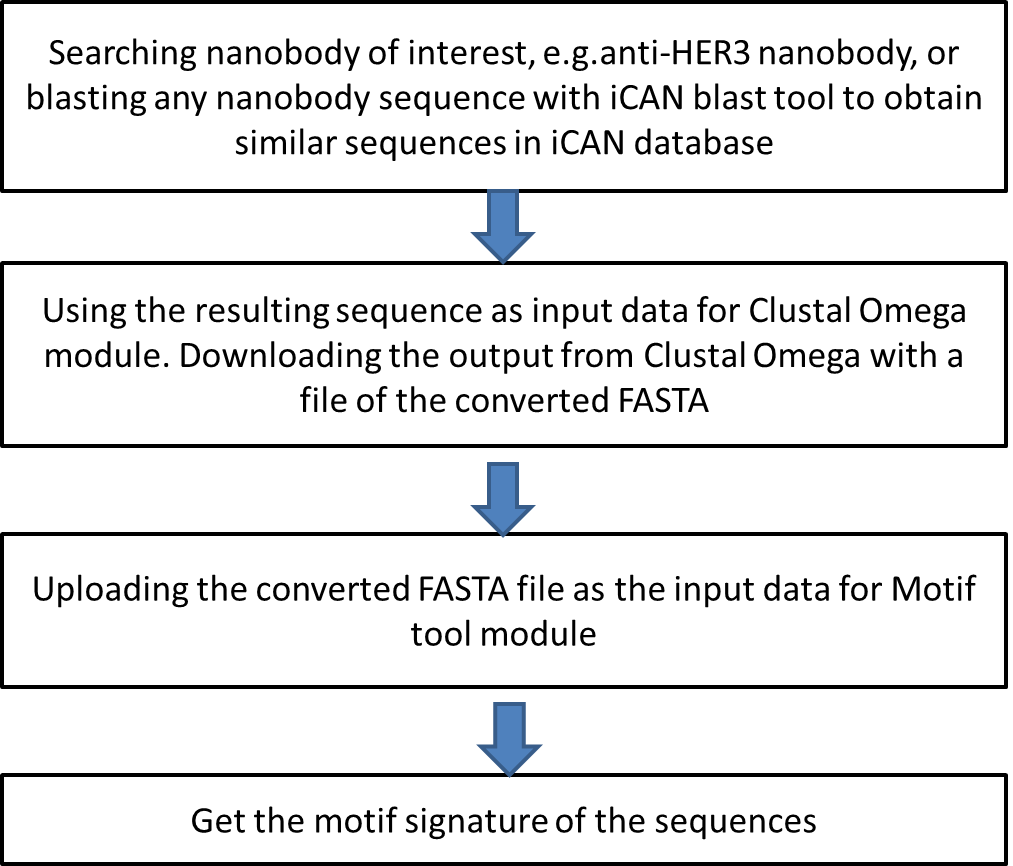
**

**Translation**

Translation tool allows the user to translate a nucleotide (DNA/RNA) sequence to a protein sequence. Users can enter a DNA or RNA sequence in FASTA format directly into the input box. The result will show 3 kinds of translated sequences from different open reading frames. At the beginning of each line of the sequence, there is a number showing the order of the first acid amino.

**Submit**

Users can submit their own nanobody data to store and analyze their nanobody. Users should submit their sequence in FASTA format and complete the required information such as users’ contact information and antigen name. We will review the sequence, give an annotation for submitted sequence and return the result to the users.

**Structure**

The structure interface shows the nanobodies whose structures are available in PDB. Users can screen nanobodies they are interested according to source organism, function and resolution and then further obtain structural information through the links to PDB.

**Prediction**

The prediction interface provides users with the CDR prediction tool. Users can input their nanobody sequences and make a prediction for CDR domains.

The following is implementation pipeline for this tool:

Map the domains to the CDR regions

**In silico defining CDR1, CDR2 and CDR3**

- - - CDR1 identification:

Search 'SC' in the 20th-26th amino acids, if not found then search for 'C' in this region

- - - - If 'SC' or 'C' is found then use 'C' as anchor, CDR1 starts at 'C'+5
      - Search 'W*R' in the 32nd-40th amino acids, if not found then search for 'W' in this region
      - If 'W*R' or 'W' is found then use 'W' as anchor, CDR1 ends at 'W'-1
      - If both end marker not found, then suppose the CDR is between 28^th^-36^th^ amino acids
    - CDR2 identification:
      - Search 'W*R' in the 32th-40th amino acids, if not found then search for 'W' in this region
      - If 'W*R' or 'W' is found then use 'W' as anchor, CDR2 starts at ‘W'+14
      - Search 'RF' in the 63nd-72th amino acids
      - If 'RF' is found then use 'R' as anchor, CDR2 ends at 'R'-8
      - If both end marker not found, then suppose the CDR is between 51^st^-60^th^ amino acids
    - CDR3 identification:
      - Search 'Y*C' in the 92nd-102nd amino acids, if not found then search for 'C' in this region
      - If 'Y*C' or 'C' is found then use 'C' as anchor, CDR3 starts at 'C'+3
      - Search 'WGQ' or ‘WG’ or ‘W’ or ‘GQ’ in the (n-14)th -(n-4)th amino acids of the protein (n = last index of the aa of the protein)
      - If 'GQ' is found then use 'G' as anchor, CDR3 ends at 'G'-2 or if 'W' is found then use 'W' as anchor, CDR3 ends at 'W'-1
      - If both end markers not found, then suppose the CDR is between (n-21)^st^-(n-10)^th^ amino acids

References

- Fridy PC, Li Y, Keegan S, Thompson MK, Nudelman I, Scheid JF, Oeffinger M, Nussenzweig MC, Fenyo D, Chait BT *et al*: A robust pipeline for rapid production of versatile nanobody repertoires. *Nature methods* 2014, 11(12):1253-1260.

**Example**

**An example of Blast**

>can_375|GFP nanobody

MQVQLVESGGALVQPGGSLRLSCAASGFPVNRYSMRWYRQAPGKEREWVAGMSSAGDRSSYEDSVKGRFTISRDDARNTVYLQMNSLKPEDTAVYYCNVNVGFEYWGQGTQVTVSSKHHHHHH

**An example of Clustal Omega**

>CAN_437

EVQLVESGGGLVQPGGSLRLSCAASGFTFGSNGMRWVRQAPGKGPEWVSSINSDGTSAFYAESVKGRFTISRDNAKNTLYLQMNSLKPEDTAVYYCTTTMNPNPRGQGTQVTVSS

>CAN_438

EVQLVESGGGLVQPGGSLRLSCAASGFTFGSNGMRWVRQAPGKGPEWVSSINSDGTSTYYAESVKGRFTISRDNAKNTLYLQMHSLKPEDTAVYYCTTTENPNPRGPGTQVTVSS

>CAN_443

EVQLVESGGGLVQPGDSLRLSCAASGFTFGSNGMRWVRQAPGKGPEWVSSINSDGTSTYYADSVKGRFTISRDNAKNTLCLQMNSLKPEDTAVYYCTTTEDPYPRGQGTQVTVSS

>CAN_585

EVQLVESGGGLVQPGGSLRLSCAASGFTFGSYDMSWVRQAPGKGPEWVSAINSGGGSTYYADSVKGRFTISRDNAKNTLYLQMNSLKPEDTAVYYCSTDLGPWIEGTMEEYEYEYWGQGTQVTVSS

>CAN_589

EVQLVESGGGLVQPGGSLRLSCVASGFTFGSYDMSWVRQAPGKRPEWVSAINSGGGSTYYADSVKGRFTISRDNAKNTLYLQMNSLKPEDTAVYYCATDEDYALGPNEYDYYGQGTQVTVSS

>CAN_1083

EVQLVESGGGLVQPGGSLRLSCAASGFPFGMYGMRWVRQAPGKGPERVSSINSDGDTTYYADSVKGRFTISRDNDENMLYLQMNSLKPEDTAVYYCATGFSDRSFAVTHKGQGTQVTVSS

**An example of Motif**

>CAN_437

EVQLVESGGGLVQPGGSLRLSCAASGFTFGSNGMRWVRQAPGKGPEWVSSINSDGTSAFYAESVKGRFTISRDNAKNTLYLQMNSLKPEDTAVYYCTTTMNPNPRG...........QGTQVTVSS

>CAN_438

EVQLVESGGGLVQPGGSLRLSCAASGFTFGSNGMRWVRQAPGKGPEWVSSINSDGTSTYYAESVKGRFTISRDNAKNTLYLQMHSLKPEDTAVYYCTTTENPNPRG...........PGTQVTVSS

>CAN_443

EVQLVESGGGLVQPGDSLRLSCAASGFTFGSNGMRWVRQAPGKGPEWVSSINSDGTSTYYADSVKGRFTISRDNAKNTLCLQMNSLKPEDTAVYYCTTTEDPYPRG...........QGTQVTVSS

>CAN_585

EVQLVESGGGLVQPGGSLRLSCAASGFTFGSYDMSWVRQAPGKGPEWVSAINSGGGSTYYADSVKGRFTISRDNAKNTLYLQMNSLKPEDTAVYYCSTDLGPWIEGTMEEYEYEYWGQGTQVTVSS

>CAN_589

EVQLVESGGGLVQPGGSLRLSCVASGFTFGSYDMSWVRQAPGKRPEWVSAINSGGGSTYYADSVKGRFTISRDNAKNTLYLQMNSLKPEDTAVYYCATDEDY....ALGPNEYDYYGQGTQVTVSS

>CAN_1083

EVQLVESGGGLVQPGGSLRLSCAASGFPFGMYGMRWVRQAPGKGPERVSSINSDGDTTYYADSVKGRFTISRDNDENMLYLQMNSLKPEDTAVYYCATGFSDR......SFAVTHKGQGTQVTVSS

**An example of Translation**

>translation example

CTGCAGGCGTCTGGGGGAGGCTTGGTGCAGCCTGGGGGTTCTCTGAGACTCTCCTGTACGTCTGGCGTCACTTTGGATACTTATGCCATAGGCTGGTTCCGCCAGGCCCCAGGGAAGGGGCGCGAGGCGGTCTCATGTATTAGTGGTGAGGATGATACCACATACTATGTAGACTCCCTGAAGGACCGATTCACCATCTCCAGAGACAACGCCAAGAACACAGTGTCTCTGCAAATGAACAGCCTAGAACCTGACGACACGGCCGTTTATTTTTGTGCAGCCTCAAGGGGAAATTTCGGTAGTGGTTGGTACTGCGGGAATGATCCACAGTATTACTACGGCATGGACTATTGGGGCAAAGGGACCCAGGTCACC

**An example of CDR prediction**

>prediction example

EVQLVESGGGLVQAGGSLRLSCAASGFSFA**DYAIG**WFRQAPGKEREGVS**CIANSEGTKYYADSAQG**RLPISSDNAKKTVYLQMDSLKPEDTAVYYCAA**LPYTICPVVVKKGAVYYGVDDY**WGKGTQVTVSS

**If you have any questions, you are welcome to contact with us (ican@seu.edu.cn).**

**Thanks for your support!**
