# Supplementary material for: Institute collection and analysis of Nanobodies (iCAN): a comprehensive database and analysis platform for nanobodies
Source: BMC Genomics. 2017 Oct 17;18:797. doi: 10.1186/s12864-017-4204-6 (PMC5646159; doi:10.1186/s12864-017-4204-6)
Supplement: Supplementary file 2 — Structure and Submit pages of iCAN website. (a) Structure page of iCAN (b) Submit page of iCAN. (PDF 639 kb) [file 12864_2017_4204_MOESM2_ESM.pdf]

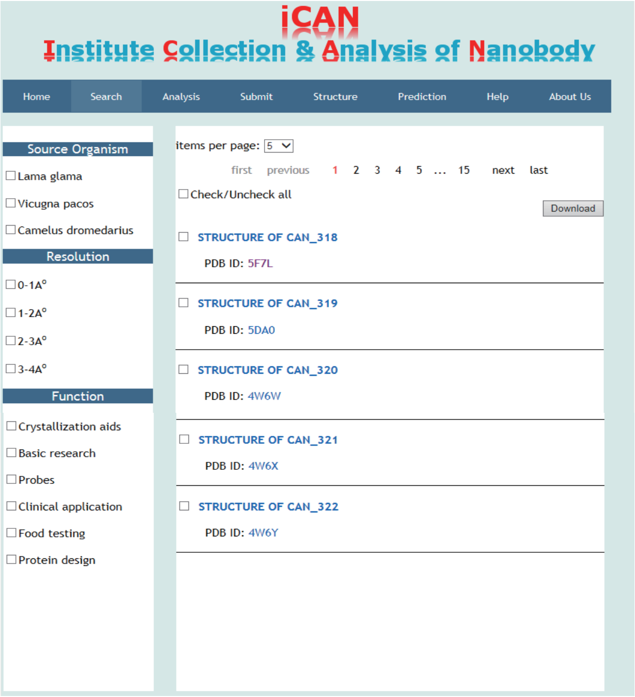
iCAN
Institute Collection & Analysis of Nanobody
Home
Search
Analysis
Submit
Structure
Prediction
Help
About Us
Source Organism
Lama glama
Vicugna pacos
Camelus dromedarius
Resolution
0-1A°
1-2A°
2-3A°
3-4A°
Function
Crystallization aids
Basic research
Probes
Clinical application
Food testing
Protein design
items per page: 5
first previous 1 2 3 4 5 … 15 next last
Check/Uncheck all
Download
STRUCTURE OF CAN_318
PDB ID: 5F7L
STRUCTURE OF CAN_319
PDB ID: 5DA0
STRUCTURE OF CAN_320
PDB ID: 4W6W
STRUCTURE OF CAN_321
PDB ID: 4W6X
STRUCTURE OF CAN_322
PDB ID: 4W6Y

a

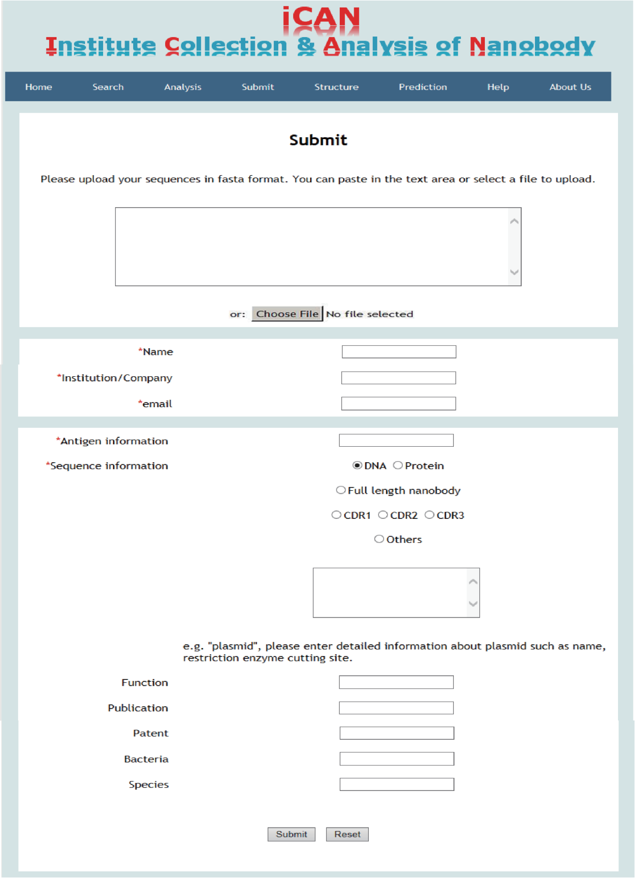
iCAN
Institute Collection & Analysis of Nanobody
Home
Search
Analysis
Submit
Structure
Prediction
Help
About Us
Submit
Please upload your sequences in fasta format. You can paste in the text area or select a file to upload.
or: Choose File No file selected
*Name
*Institution/Company
*email
*Antigen information
*Sequence information
DNA Protein
Full length nanobody
CDR1 CDR2 CDR3
Others
e.g. "plasmid", please enter detailed information about plasmid such as name, restriction enzyme cutting site.
Function
Publication
Patent
Bacteria
Species
Submit
Reset

b
