# Supplementary figures and images for: Institute collection and analysis of Nanobodies (iCAN): a comprehensive database and analysis platform for nanobodies
Source: BMC Genomics. 2017 Oct 17;18:797. doi: 10.1186/s12864-017-4204-6 (PMC5646159; doi:10.1186/s12864-017-4204-6)

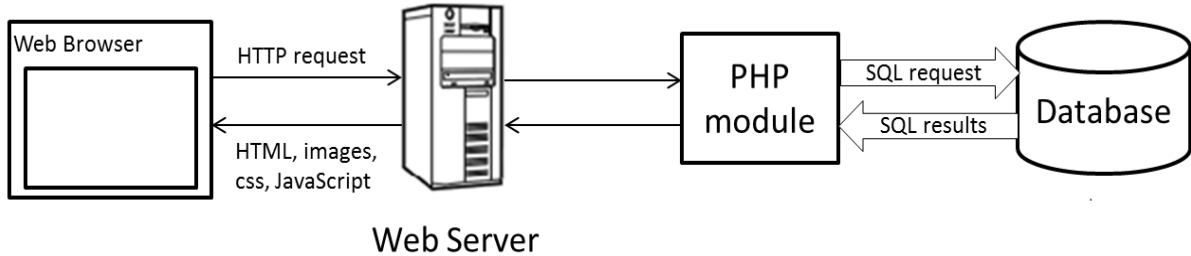

Supplement: Supplementary file 3 — The workflow of iCAN website. After the user’s Web browser sends a HTTP query, the required HTML pages are returned in response to user’s query. (PDF 294 kb) [file 12864_2017_4204_MOESM3_ESM.pdf]
